# Three-Dimensional Photoacoustic Endoscopic Imaging of the Rabbit Esophagus

**DOI:** 10.1371/journal.pone.0120269

**Published:** 2015-04-15

**Authors:** Joon Mo Yang, Christopher Favazza, Junjie Yao, Ruimin Chen, Qifa Zhou, K. Kirk Shung, Lihong V. Wang

**Affiliations:** 1 Optical Imaging Laboratory, Department of Biomedical Engineering, Washington University in St. Louis, Saint Louis, Missouri, United States of America; 2 National Institutes of Health Ultrasonic Transducer Resource Center, Department of Biomedical Engineering, University of Southern California, Los Angeles, California, United States of America; Institute of Automation, Chinese Academy of Sciences, CHINA

## Abstract

We report photoacoustic and ultrasonic endoscopic images of two intact rabbit esophagi. To investigate the esophageal lumen structure and microvasculature, we performed *in vivo* and *ex vivo* imaging studies using a 3.8-mm diameter photoacoustic endoscope and correlated the images with histology. Several interesting anatomic structures were newly found in both the *in vivo* and *ex vivo* images, which demonstrates the potential clinical utility of this endoscopic imaging modality. In the *ex vivo* imaging experiment, we acquired high-resolution motion-artifact-free three-dimensional photoacoustic images of the vasculatures distributed in the walls of the esophagi and extending to the neighboring mediastinal regions. Blood vessels with apparent diameters as small as 190 μm were resolved. Moreover, by taking advantage of the dual-mode high-resolution photoacoustic and ultrasound endoscopy, we could better identify and characterize the anatomic structures of the esophageal lumen, such as the mucosal and submucosal layers in the esophageal wall, and an esophageal branch of the thoracic aorta. In this paper, we present the first photoacoustic images showing the vasculature of a vertebrate esophagus and discuss the potential clinical applications and future development of photoacoustic endoscopy.

## Introduction

Diseases of the epithelium, such as Barrett’s esophagus [1], can be precursors to high-grade dysplasia and adenocarcinoma; 85% of all cancers originate in the epithelium [2,3]. It is well known that early detection of esophageal adenocarcinoma improves treatment outcomes. Consequently, considerable effort has focused on improving the endoscopic surveillance programs employed to routinely monitor patients with Barrett’s esophagus [1,4,5]. Currently, endoscopic ultrasound (EUS) [6–8] is the dominant clinical tomographic endoscopic tool used to diagnose many diseases in the gastrointestinal (GI) tract. However, EUS is unable to provide sufficiently informative microscopic images, such as of the microvasculature, which has been shown to be diagnostic of Barrett’s esophagus [9–13]. Well-known optical endoscopic techniques, such as endoscopic optical coherence tomography [14–21] and confocal endoscopy [13,22–25], have proven capable of improving the diagnostic accuracy of Barrett’s esophagus [15–17,19,21] by yielding high image contrast and micron-level spatial resolution. However, optical coherence tomography relies on the Doppler principle to render images of blood vessels [14,18], which requires sufficient blood flow speed and thus limits sensitivity. Confocal endoscopy requires fluorescence labeling of an exogenous contrast agent to image blood vessels [13,25], and its point-scanning mechanism does not yield depth-resolved signals. Additionally, endoscopic polarized scanning spectroscopy [2,3,26,27] has recently shown that spectroscopic imaging can provide additional diagnostic information in screening for the disease. However, this technique has not demonstrated the capability to image vasculature.

Given the deficiencies of the current imaging modalities, a newly-emerging endoscopic technique, called photoacoustic endoscopy (PAE) [28–30], could be an important complement in diagnosing such GI tract diseases because it is well-equipped to provide high resolution microvasculature imaging with rich spectral and functional information of the tissue [29]. As a step toward clinical application of PAE, in 2012 [29], we demonstrated *in vivo* three-dimensional, trans-esophageal photoacoustic (PA), and ultrasonic (US) imaging of organs and major blood vessels in the rabbit mediastinum using a 3.8-mm diameter PA endoscope. However, strong motion artifacts, primarily caused by respiratory motion, coupled with an insufficient axial imaging frame rate (∼4 Hz), prevented *in vivo* acquisition of high-resolution, three-dimensional vasculature images in the esophagus. Consequently, the presented mediastinum images suffered substantial image resolution degradation from the application of surface-alignment and spatial filtering algorithms, which were necessary to produce continuous volumetric images and artificially mitigate these motion artifacts.

In this study, to further investigate this technique’s potential, we imaged *in vivo* and *ex vivo* two intact rabbit esophagi, and correlated the images with histology. First, to show the clinical potential of our endoscopic imaging system, we imaged the two esophagi *in vivo*. Subsequently, we reimaged the esophagi *ex vivo*. By imaging *ex vivo*, the aforementioned complications from respiratory motion artifacts were easily resolved, and such an *ex vivo* imaging strategy is commonly employed when evaluating the capability of new technologies to image anatomical features for the first time [20,24,31]. Through *ex vivo* imaging, we acquired three-dimensional PA images showing the vasculature and lumenal structure of intact rabbit esophagi, utilizing the full vascular resolving power of our PAE system. These results could be a valuable image reference for future PAE technology development. To date, there have been no published reports of high-resolution PA endoscopic images of the microvasculature of the esophagus, either *in vivo* or *ex vivo*. The results from this study demonstrate the current capability of our PAE to resolve esophageal microvasculature and the potential of PAE to justify further development, including increased imaging speed and respiratory motion mitigation strategies. Here we present the first three-dimensional PA images acquired from the esophagi of two rabbits and discuss the potential clinical applications and future development of PAE.

## Materials and Methods

### Dual-modality PAE-EUS imaging system

**Fig 1:**
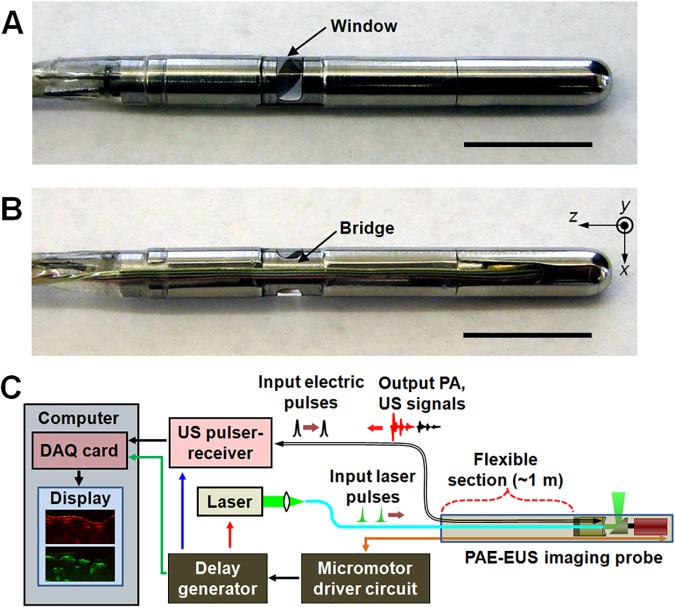
Dual-modality PAE-EUS imaging probe and peripheral systems. (**A**, **B**) Photos showing the 3.8-mm diameter PAE-EUS probe’s imaging window (**A**) and bridge (**B**) sections. The inner cavity of the endoscope is filled with de-ionized water to provide acoustic coupling between the probe’s imaging window and the US transducer. In (**B**), +*z* is defined along the endoscope axis and pullback direction. Scale bars, 1 cm. (**C**) Block diagram showing the endoscope and peripheral systems. The peripheral systems are synchronized to the TTL signals provided by the micromotor driver circuit, and triggered by the delay generator with different delays.

For these experiments, we utilized the 3.8-mm diameter side-scanning PAE-EUS probe (**Fig. 1A and 1B**) and peripheral systems (**Fig. 1C**) reported in our recent paper [29]. The endoscopic system enables simultaneous PA and US imaging of internal organs. The endoscopic probe’s 3.8-mm diameter and working distance (∼0.5 mm from the probe’s surface) are suitable for imaging rabbit esophagus, which has a comparable diameter. By employing a tunable dye laser (Cobra HRR, Sirah), pumped by a solid-state, diode-pumped Nd:YLF laser (INNOSLAB IS811-E, EdgeWave) and an US pulser-receiver (5072PR, Panametrics) (**Fig. 1C**), we could acquire co-registered PA and US images at a B-scan frame rate of ∼4 Hz. We imaged at a 584 nm wavelength (∼10 ns pulse width) with a laser energy of ∼0.3 mJ/pulse (optical fluence: ∼10 mJ/cm^2^) to produce PA image contrast proportional to the total hemoglobin concentration. To record coregistered PA and US images, we excited and acquired PA and US A-line signals in alternation, with each signal offset by 30 μs from the previous signal, at every 1.42° (0.98 ms at 4 Hz) of the mirror’s constant rotation.

The endoscopic system’s radial field-of-view (FOV) is ∼7 mm (i.e., ∼18 mm in diameter including the space occupied by the probe), and its angular FOV is 270°. A portion of the angular FOV is blocked by the electric wires connected to the micromotor that is installed in the rigid distal section of the probe (**Fig. 1B**). The experimentally determined PA and US resolutions of the focused US transducer (∼36 MHz, 65% fractional bandwidth, LiNbO_3_) in the focal zone were respectively ∼55 μm and ∼30 μm in the radial (or axial) direction, and ∼80 μm and ∼60 μm in the transverse direction [29]. All the resolution measurements were performed outside the probe housing, with the device only partially assembled [29]. Volumetric data sets were acquired by recording sequential A-line data during the constant rotational motion of the mirror and mechanical pullback of the probe, which resulted in a helical scanning with a pitch of ∼40 μm. To record the detected PA and US signals, we utilized a 12-bit data acquisition (DAQ) card (200 MHz, NI PCI-5124, National Instruments). More detailed information on the imaging system is available elsewhere [29].

### Rabbit esophagus imaging

With the endoscopic system, we imaged the intact esophagi of two adult New Zealand white rabbits (∼4 kg, ∼6-month-old, Myrtle’s Rabbitry) *in vivo* and *ex vivo*. The rabbits were fasted, beginning ∼12 hr before the experiments, to reduce the likelihood of ingesta in the upper GI tracts. Prior to endoscopic imaging, we anesthetized the rabbit with 35–50 mg/kg of ketamine and 5–10 mg/kg of xylazine (IM). While anesthetized, the rabbit was intubated and supplied with maintenance gas for anesthesia (1.5–3.0% isoflurane). We inserted an endotracheal tube cuff into the trachea and inflated it to prevent aspiration of water into the lung. We placed the rabbit on an inclined stage (∼10°) in the supine position. Just before probe insertion, we filled the esophagus with water using an enteral feeding syringe connected to a rubber feeding tube (8–12 F). The water provided the necessary acoustic coupling and functioned as a lubricant during the imaging procedure. After filling the esophagus with water, we inserted the endoscopic probe through the mouth and advanced it approximately 25–30 cm, to the point at which the probe could no longer be gently advanced. Simultaneous PA and US imaging were immediately initiated. During image acquisition, the probe was slowly and mechanically pulled out of the esophagus over a ∼12 cm range, using a motorized translation stage at a speed of ∼160 μm/s. About 3000 B-scan slices with a longitudinal spacing of ∼40 μm were acquired for each imaging mode. Throughout the experiment, we continuously monitored the rabbit’s anesthesia level and vital signs. After the *in vivo* experiment, we euthanized the rabbit by injection of an overdose of sodium pentobarbital (150 mg/kg) into the marginal ear vein. We then imaged the esophagus *ex vivo* with the same procedure as before. After finishing the imaging experiment, we dissected the animal and collected samples from the esophagus for histological analysis, which was compared with PA and US images. All procedures in the animal experiments followed protocols approved by the Institutional Animal Care and Use Committee at Washington University in St. Louis.

## Results and Discussion

### *In vivo* imaging results

**Fig 2:**
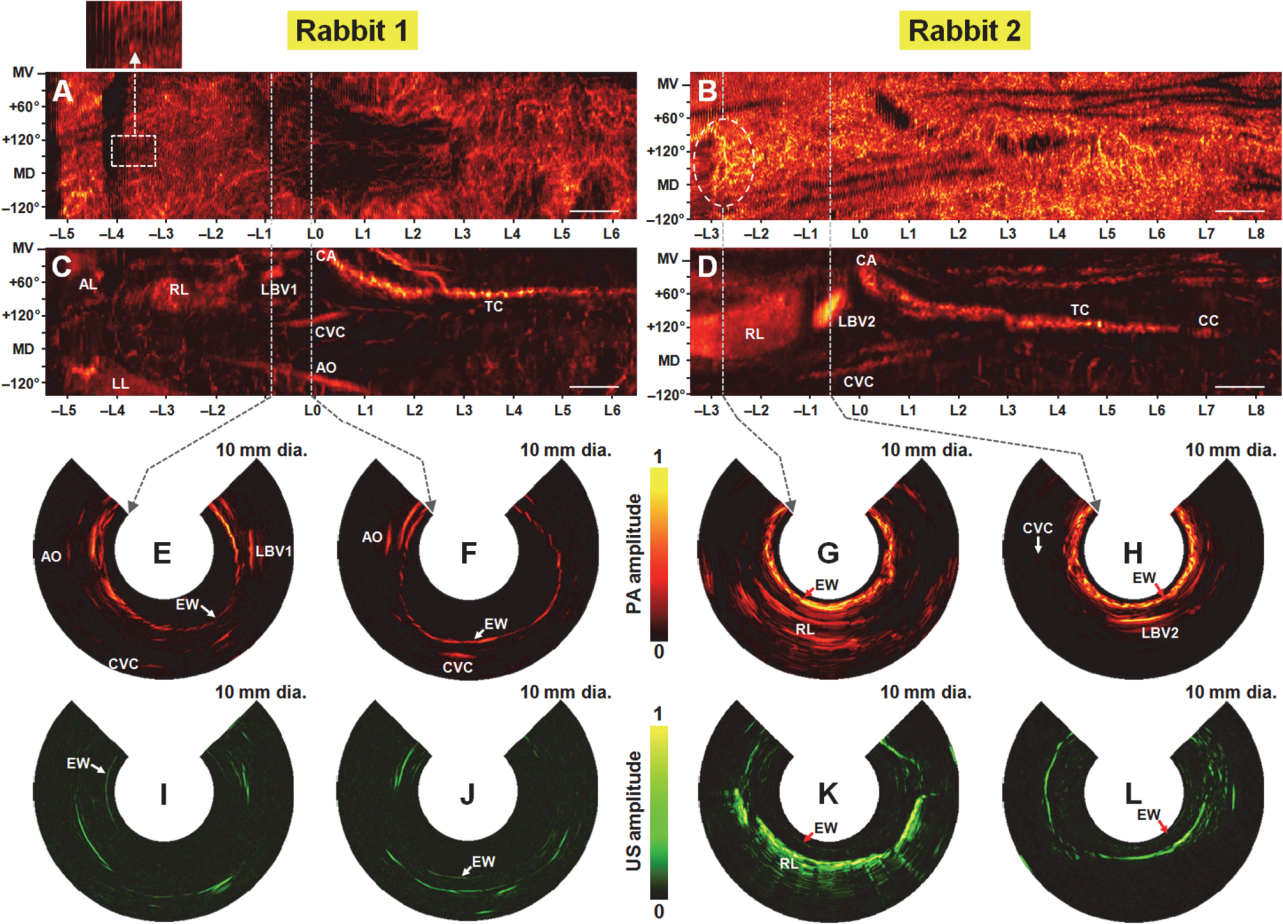
Coregistered PAE-EUS pseudocolor images acquired from rabbit esophagi *in vivo*. (**A**, **B**) Normalized PA-RMAP images of two rabbit esophagi, showing the total hemoglobin distribution (views from the inside of the esophagus). In each image, the left- and right-hand sides correspond to the lower and upper esophagus, respectively, and the imaged area covers a 270° angular FOV (vertical) and a ∼12 cm long pullback distance (horizontal). (**C**, **D**) Normalized PA-RMAP images of the mediastina for the two rabbits, with the esophageal signals excluded during the RMAP construction. AL, accessory lobe; LL, left lobe; RL, right lobe; CA, carina; TC, trachea; CVC, caudal vena cava; AO, aorta; CC, cricoid cartilage; LBV1 & LBV2, large blood vessels. In (**A**)–(**D**), the approximate mid-ventral (MV) position and angular displacement from the MV position are marked along the vertical axis; the positive and negative values correspond to the right and left sides of the animal, and MD denotes the mid-dorsal position. (**E**–**H**) PAE and (**I**–**L**) EUS B-scan images of the esophagi chosen from the marked positions. In the case of (**B**), we omitted the marking inside the dashed circle to clearly show the structures in the circled region. Each image covers a 10 mm diameter FOV. EW, esophageal wall; LBV1 & LBV2, large blood vessels; AO, aorta; CVC, caudal vena cava; RL, right lobe of the lung. All PA and US signal amplitudes are mapped on a linear scale. Scale bars, 10 mm (horizontal only).

For both rabbits, we were able to detect PA signals from several major organs in the mediastinal regions. However, the appearance of these organs and neighboring vasculature differed between the two rabbits. To illustrate this variability, we present the images of both animals in **Fig. 2**. **Fig. 2A and 2B** are esophageal PA radial-maximum amplitude projection (RMAP) images. **Fig. 2C and 2D** are mediastinal PA-RMAP images, and **Fig. 2E-2L** are PA and US cross-sectional (or B-scan) images chosen at the marked positions. To produce these images, we first applied the Hilbert transform to the B-scan images, which yields images based on the envelopes of the recorded bipolar acoustic signals. Esophageal signals were then separated from the mediastinal signals based on the depth and structure of the signal in the B-scan image; however, accurate separation was complicated by motion artifacts. The two-dimensional RMAP images were generated by “unrolling” the three-dimensional image and projecting the maximum signal value within the depth range for that image (i.e., ∼0.4 mm for esophageal RMAP images and ∼6.6 mm for mediastinal RMAP images). Note that, to more clearly depict structures in the esophagus, the B-scan images in **Fig. 2E-2L** show only a 3.1 mm radial depth (i.e., 10 mm diameter FOV) which constitutes only a portion of the depth of the mediastinal regions; however, the B-scan images contain the major structures depicted in the mediastinal RMAP images. In each RMAP image, the longitudinal and transverse locations of the esophagus are marked in the horizontal and vertical axes, respectively, where the longitudinal location of the carina (CA) is set at as L0.

As shown in **Fig. 2E-2H**, we were able to acquire cross-sectional PA images of blood vessels distributed in the wall of the esophagi and adjacent mediastinal regions that are virtually free of motion artifacts. We also acquired corresponding, coregistered US images (**Fig. 2I-2L**). However, the PA- and US-RMAP images suffer from motion artifacts. With an axial image acquisition speed of 4 Hz and a rabbit respiration rate of ∼0.25 Hz, RMAP images were acquired over many respiration cycles, which led to discontinuous vasculature maps as demonstrated by **Fig. 2A and 2B** that display the raw Hilbert-transformed data without additional filtering. In the case of **Rabbit 1**’s esophageal RMAP image (**Fig. 2A**), vascular structures superior to the lungs are more accurately mapped, whereas in regions nearer to the lungs (i.e., from—L5 to L0) the image is more corrupted by respiration. The magnified image shows a periodic image distortion corresponding to the breathing cycle of ∼0.25 Hz. In the case of **Rabbit 2**’s esophageal RMAP image (**Fig. 2B**), the motion artifacts (more specifically transverse shear motion) are even stronger than those of **Rabbit 1**, and thus the overall vasculature pattern is not as clear as in **Rabbit 1**’s image. However, several prominent structures, which are assumed to be a single network of several blood vessels, were imaged at the lower esophagus (see the dashed circle) and are discussed later. Esophageal US-RMAP images were equally corrupted by respiratory motion; however, the relative thinness of the esophagi compared to motion-based displacements and the low image contrast of mucosa preclude the generation of decipherable images. We present the esophageal US-RMAP images in **S1 Fig**.

Without additional imaging processing, strong motion artifacts also precluded clear visualization of the organs and blood vessels in the mediastinal images (**Fig. 2C and 2D**). With the application of the spatial filtering algorithm utilized in a previous study [29], motion artifacts were artificially corrected at the expense of spatial resolution. Many fine structures and small vessels were blurred through this process; however, many larger anatomic structures became clearly distinguishable. As shown in the images, the two major organs, the lung (LL: left lobe, RL: right lobe) and trachea (TC), were imaged in both animals, as previously demonstrated [29]. The caudal vena cava (CVC) and aorta (AO) are also identified in **Rabbit 1**’s mediastinal image (**Fig. 2C**). A very large blood vessel (LBV1), which is assumed to be a portion of the pulmonary vascular system, was detected between the right lobe (RL) and carina (CA) for the first time; a similar large blood vessel (LBV2) also appeared in **Rabbit 2**’ mediastinal image (**Fig. 2D**) with an even larger diameter. For the two PA mediastinal RMAP images (**Fig. 2C and 2D)**, we could acquire corresponding US-RMAP images as presented in **S1 Fig.** by applying the same spatial filtering algorithm.

### *Ex vivo* imaging results

**Fig 3:**
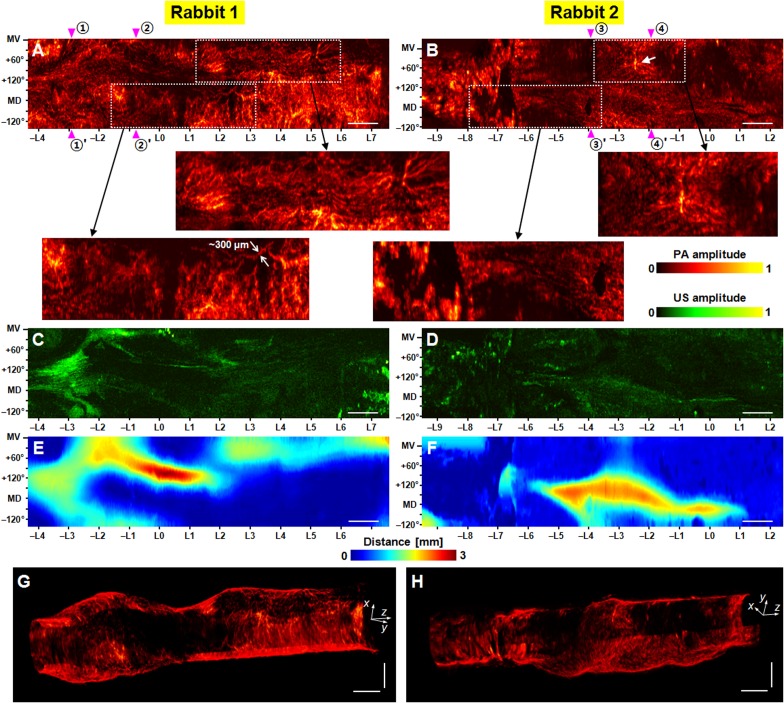
Coregistered PAE-EUS pseudocolor *ex vivo* images showing vasculature and tissue density distributions of rabbit esophageal walls. (**A**, **B**) Normalized PA-RMAP images of two rabbit esophagi, showing the total hemoglobin distribution (views from the inside of the esophagus). In each image, the left- and right-hand sides correspond to the lower and upper esophagus, respectively, and the imaged area covers a 270° angular FOV (vertical) and a ∼12 cm long pullback distance (horizontal). In (**B**), the arrow indicates an esophageal branch of the thoracic aorta. Panels in the second row are magnified images of the rectangular zones (PA signal amplitudes were renormalized). (**C**, **D**) Corresponding normalized US-RMAP images of the full area PA-RMAP images shown in (**A**) and (**B**), respectively. (**E**, **F**) Esophagus surface distance maps measured from the 3.8-mm diameter endoscopic probe’s surface, using the PA volumetric data presented in (**A**) and (**B**), respectively. In (**A**)–(**F**), the approximate mid-ventral (MV) position and angular displacement from the MV position are marked along the vertical axis; the positive and negative values correspond to the right and left sides of the animal, and MD denotes the mid-dorsal position. (**G**, **H**) Three-dimensionally rendered PA endoscopic images of the two rabbit esophagi. **S1 Video** shows more detailed structures for image (**G**). All PA and US signal amplitudes are mapped on a linear scale. Scale bars, 10 mm (horizontal) and 5 mm (vertical).

While the *in vivo* imaging experiments suffered from motion artifacts, the *ex vivo* imaging experiments enabled the acquisition of continuous three-dimensional maps of esophageal vasculatures and thus more convincing image interpretation. To show the effect of the motion artifacts on the image quality degradation, we present the *in vivo* and *ex vivo* PA and US esophageal RMAP images in **S2 Fig**. and present only the *in vivo* and *ex vivo* PA-RMAP images in **S3 Fig.** Also with *ex vivo* imaging, we could acquire reliable esophageal US-RMAP images for the two animals, which was not possible in the *in vivo* experiment. Thus in **Fig. 3**, we present the coregistered PA and US *ex vivo* esophageal images together. First, in **Fig. 3A and 3B**, we present PA-RMAP images of the two esophagi, showing the entire scanned areas along with several magnified images of selected regions of interest. **Fig. 3C and 3D** are corresponding US-RMAP images, respectively. The PA data sets showed much stronger surface signals than the US data, clearly delineating the esophagus surface. Thus, using the PA data sets, we mapped the esophagus surface-to-endoscope distance, shown in **Fig. 3E and 3F**. Based on the acquired esophagus surface distance maps and the calculated wall thickness of about ∼400 μm, we were able to extract the esophagus signal accurately and produce PA- and US-RMAP images (**Fig. 3A**–**3D**) of the esophagus only.

**Fig 4:**
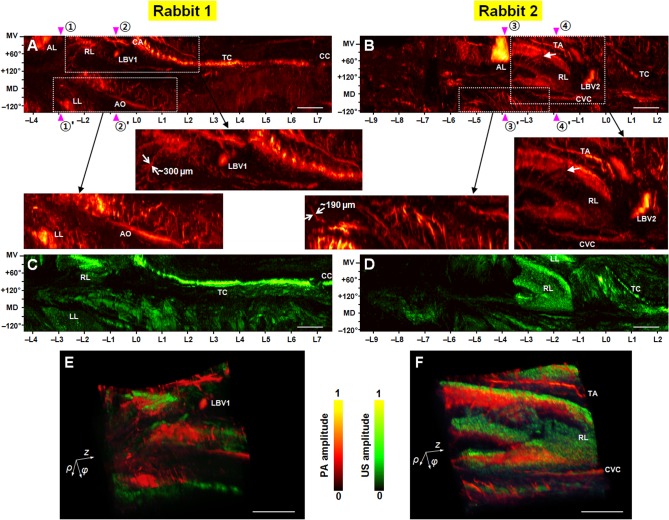
Coregistered PAE-EUS pseudocolor *ex vivo* images showing the vasculatures and tissue density distributions of rabbit mediastina. (**A**, **B**) Normalized PA-RMAP images of the mediastina for the two rabbits (views from the inside of the esophagus), with the esophageal signals excluded during the RMAP construction. In each image, the left- and right-hand sides correspond to the lower and upper esophagus, respectively, and the imaged area covers a 270° angular FOV (vertical) and a ∼12 cm long pullback distance (horizontal). Panels in the second row are magnified images of the rectangular zones (PA signal amplitudes were renormalized). (**C**, **D**) Corresponding normalized US-RMAP images of the full area PA-RMAP images shown in (**A**) and (**B**), respectively. AL, accessory lobe; LL, left lobe; RL, right lobe; CA, carina; TC, trachea; AO, aorta; CVC, caudal vena cava; CC, cricoid cartilage; TA, thoracic aorta; LBV1 & LBV2, large blood vessels. In (**A**)–(**D**), the approximate mid-ventral (MV) position and angular displacement from the MV position are marked along the vertical axis; the positive and negative values correspond to the right and left sides of the animal, and MD denotes the mid-dorsal position. (**E**, **F**) Three-dimensionally rendered, co-registered, PAE-EUS images taken from the longitudinal section approximately from—L3 to L0 of (**A**) and (**B**), with a 270° angular FOV. In each image, the *ρ*-axis corresponds to the radial depth, the *φ*-axis corresponds to the scanning mirror’s rotational direction, and the *z*-axis corresponds to the pullback direction. Scale bars, 10 mm (horizontal only).

The PA-RMAP images (**Fig. 3A and 3B**) show clear vascular maps of the esophagi. **Rabbit 1**’s image (**Fig. 3A**) shows a densely-distributed vascular network with tortuosity variation over the scanned area. From the calculated esophagus wall thickness of ∼400 μm and the plotted blood vessels distributed in the wall, we could estimate blood vessel diameter based on the PA esophagus surface distance maps (**Fig. 3E and 3F**). Apparent blood vessel diameters in the magnified PA-RMAP images are less than 300 μm. **Rabbit 2**’s image (**Fig. 3B**) shows similar vascular structures beginning near the middle esophagus region (i.e., near position—L6). Note that the probe was inserted ∼5 cm deeper into **Rabbit 2** than into **Rabbit 1**. In the lower ∼3 cm region (i.e., left region of—L7) of the imaged esophagus of **Rabbit 2**, clear vascular networks are not visible. Rather, we see a large region of diffuse signals. We speculate that these diffuse signals are from capillary beds, which cannot be resolved by the current endoscope. Another notable structure in this image is the blood vessel network (marked with the arrow) which appeared at the same location of the marked blood vessel (i.e., inside the dashed circle) in **Fig. 2B**. Matching regions in the *in vivo* and *ex vivo* esophageal RMAP images was achieved through visual co-registration of anatomical landmarks in the *in vivo* and *ex vivo* mediastinal RMAP images. The *ex vivo* mediastinal RMAP images are shown in **Fig. 4**. After the image interpretation, we concluded that this major large vessel is an esophageal branch of the thoracic aorta, a feeding blood vessel, which branches out from the aorta to supply blood to the esophagus (see Fig. 3 in reference [32]). Detection of this blood vessel is important because the visualization of such feeding blood vessels is crucial when performing an esophagectomy, as discussed in references [32–34]. For a three-dimensional appreciation of the imaged vascular structures, we present volume-rendered PA images of the esophagi in **Fig. 3G** (**S1 Video**) and **3H.**

Although the PA images provide clear maps of blood vasculature, the US images (**Fig. 3C and 3D**) do not, likely due to the small vessel diameters. In fact, throughout the experiments, the simple pulse-echo US imaging mode of the endoscope did not provide any distinctive blood vessel profiles; whereas, PAE demonstrated superior angiographic imaging capability. The US image contrasts are primarily related to the orientation of the wrinkled esophageal walls’ surface to the acoustic pulses; the echogenicity distributions appeared to have close relations with the esophagus surface distance maps (**Fig. 3E and 3F**). However, it should be noted that the high echoic regions do not directly correspond to high tissue density regions because the images were acquired using a single element-based focused US transducer and a typical tomography reconstruction algorithm, which compensates for acoustic attenuation, was not used to generate the images.

As shown in **Fig. 4**, the *ex vivo* experiment also enabled more clear three-dimensional visualization of vasculature extending into the mediastinal regions of the esophagi. As with the *in vivo* experiment, overall shapes and locations of several major organs, such as the accessory (AL), left (LL), and right (RL) lobes of the lung, as well as the carina (CA), and trachea (TC) are plainly visible in these *ex vivo* RMAP images. However, in the *ex vivo* imaging experiment, we could visualize those organs’ peripheral blood vessels absent the spatial resolution degradation caused by the implementation of the spatial filtering algorithm applied to the *in vivo* images (**Fig. 2C and 2D**). Apparent diameters of these vessels are as low as 190 μm. In the **Rabbit 1**’s PA-RMAP image (**Fig. 4A**), the caudal vena cava (CVC) was not imaged. However, the aorta (AO) and the large blood vessel (LBV1) were clearly visualized in this image. Also, the approximate location of the cricoid cartilage (CC) is shown because the imaged area was slightly shifted to the upper esophageal region.

In the case of **Rabbit 2**’s PA-RMAP image (**Fig. 4B**), the trachea (TC) signals appeared to be weaker than those in the *in vivo* image—note that this *ex vivo* image was acquired at a deeper region of the esophagus than the *in vivo* image, by ∼5 cm. The caudal vena cava (CVC) as well as the accessory (AL) and right (RL) lobes of the lung were clearly imaged and along with peripheral vasculatures. More importantly, a blood vessel network, which is assumed to contain the mother blood vessel that feeds the esophageal branch vessel (shown in **Fig. 3B**) of the thoracic aorta, is displayed in the magnified mediastinal image (the arrow marks the same angular and longitudinal location of the arrow shown in **Fig. 3B**). To better display the vasculature details in the mediastinal regions of the two animals, we present volume rendered, coregistered PA and US mediastinum images taken from the longitudinal section approximately from—L3 to L0 in **Fig. 4E and 4F**, respectively. In **S4 Fig.**, which corresponds to **Fig. 4F**, we artificially marked the mother blood vessel network. In **S2** and **S3 Videos**, we present a set of serial PA- and US-RMAP images of **Rabbit 2** produced by changing the depth of removed surface signals, and a volume-rendered image of the mediastinum corresponding to **Fig. 4B and 4D,** respectively.

As shown in the *in vivo* (**Fig. 2C and 2D**) and *ex vivo* (**Fig. 4A and 4B**) PA mediastinal RMAP images, overall anatomical structures in the imaged volumes were similar for the two animals. However, not all of the organs were imaged for each animal, probably due to differences in the positions of the organs, which can vary with the body habitus of the animal and imaging posture.

### Histological comparison of *ex vivo* B-scan images

**Fig 5:**
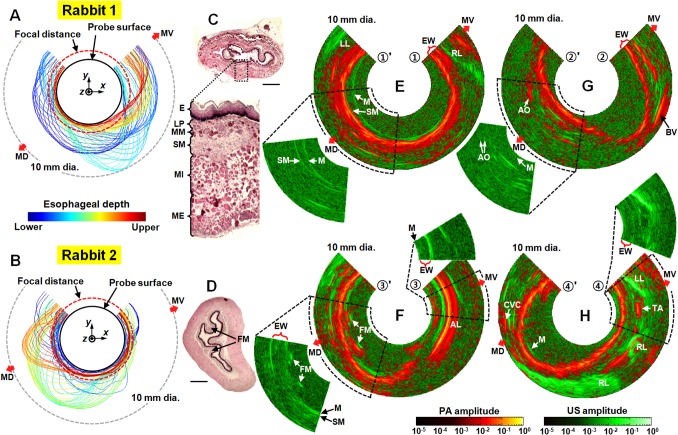
Coregistered PAE-EUS pseudocolor *ex vivo* images showing cross-sections of rabbit esophageal walls. (**A**, **B**) Esophageal inner surface variation graphs showing the radial position of the inner surface relative to the probe along the longitudinal location of the esophagus. MV, mid-ventral; MD, mid-dorsal. (**C**, **D**) Histologic images (H&E stain) of the esophagi. E, stratified squamous epithelium; LP, lamina propria; MM, muscularis mucosa; SM, submocosa; MI, muscularis interna; ME, muscularis externa; FM, folded mucosa. Scale bars, 1 mm. (**E**–**H**) Combined PA and US B-scan images chosen from the marked positions in **Figs. 3(A, B)** and **4(A, B)**. Each image covers a 10 mm diameter FOV, and shows the time-gain compensated PA and US signals. EW, esophageal wall; RL, right lobe of the lung, LL, left lobe of the lung; AO, aorta; TA, thoracic aorta; CVC, caudal vena cava; FM, folded mucosa.

To show PA image features within the esophageal wall, we present coregistered PA and US cross-sectional (B-scan) images of the two esophagi processed from the *ex vivo* data sets, along with two histologic images of samples harvested from the animals (**Fig. 5**). First, in **Fig. 5A and 5B**, we present two graphs showing the contour variations of the esophageal walls, indicating the distance of the inner surface of the esophagus from the PAE probe at longitudinal locations shown by the color bar (these graphs were plotted using the data shown in **Fig. 3E and 3F**). As shown, the imaged esophageal lumen diameters of the two rabbits ranged mostly between 4 mm and 7 mm values, which are close to the focal distance (located ∼5 mm from the image center) of the endoscope; thus the 3.8-mm diameter endoscope is suitable for rabbit esophagus imaging. The two histologic images (**Fig. 5C and 5D**) acquired from the mid-esophagus also showed similar lumen diameters (both are presented in the same scale as **Fig. 5A and 5B**); however their thicknesses changed during the histological fixation process. In **Fig. 5E-5H**, the presented coregistered PA and US cross-sectional images were cropped at 10 mm image diameter for display purposes because the esophageal lumen diameters were less than 10 mm along the entire length of the esophagi (**Fig. 5A and 5B**). The presented PA and US cross-sectional images (**Fig. 5E-5H**) were selected from the marked positions in **Fig. 3A and 3B** and **Fig. 4A and 4B**; see the locations ①–①' and ②–②' for **Rabbit 1**, and ③–③' and ④–④' for **Rabbit 2**. To more clearly display features at greater distances from the PAE probe, we applied time-gain compensation to the PA and US images.

Although the US images sometimes showed clear boundaries of the mucosal and submucosal layers (see the separate US images at the left-hand sides of **Fig. 5E and 5F**), overall US signal intensities of those layers were very weak, as shown in the separate US image at the right-hand side of **Fig. 5F**. Conversely, PA images provided a clear boundary of the esophageal wall over the entire length of the esophagus. This clear boundary is the result of strong PA signals generated from the densely distributed capillaries in the submucosal layers. Consequently, we utilized the PA data for delineating the inner boundary of the esophagi (**Fig. 3E and 3F**, and **Fig. 5A and 5B**). In clinical US endoscopy, it is straightforward to identify the mucosal and submucosal layers because they generate distinctive echo signals with adequate intensity [6–8]. However, in our experiment, US signals from those layers were very weak because we employed a focused US transducer and set the gain (dynamic range) of the signal amplifier to optimize registration of signals from other hyper-echoic regions, such as the trachea. The inner boundary of the esophageal walls appeared strongly in US images only when they were located near the focal point of the transducer and their surface was perpendicular to the acoustic axis.

Using the PA signals in **Fig. 5E-5H**, we could estimate the wall thickness of the two esophagi to be ∼400–600 μm, slightly thinner than that in the histologic images (**Fig. 5C and 5D**). These slight discrepancies are likely due to distortion of the tissue during the histological fixation. Also, through inspection of the PA-US overlapped images, we could better identify the inner boundary of the esophageal wall (submucosal layer) as well as the locations of major organs and blood vessels surrounding the esophagi, such as the accessory lobe (AL) (**Fig. 5F**), aorta (AO) (**Fig. 5G**), and caudal vena cava (CVC) (**Fig. 5H**). Across the entire sets of B-scan images acquired from the two rabbits, the gaps between the mucosal and submucosal layers were generally identified to be very narrow (less than ∼100 μm). However, wide separations were observed in some regions (see the left inner wall of **Fig. 5E**), and these regions correspond to the high echoic regions of **Fig. 3C**. To better understand the underlying reasons for these differences in separations, more imaging studies and a more thorough investigation are needed.

Based on the acquired images, PAE has demonstrated several unique attributes which may be clinically beneficial for esophageal and periesophageal disease diagnosis and treatment. As shown in the presented PA images, the major benefit of PAE is that it enables the label-free visualization of the microvasculature in the esophagus (**Figs. 2** and **3**), whose importance for Barrett’s esophagus diagnosis has been discussed in many reports [9–13]. Also, the PA images provide additional morphologic information of target tissues, which promotes their anatomic identification in concert with EUS (**Fig. 5**). With the employed endoscopic probe, we could visualize blood vessels as small as 190 μm in diameter, which exceeds the current capability of EUS technique, and we demonstrated vasculature mapping of nearly the entire region of the esophagus in a scanning time of ∼10 minutes. Although a contrast-enhanced version of EUS technique, called endoscopic Doppler ultrasonography, is also capable of imaging blood vessels, its sensitivity is much lower than that of PAE, and thus in the clinic it is limited to imaging large blood vessels only. Another benefit of PAE for esophageal imaging is that it can visualize vascular networks extending into the neighboring mediastinal regions (**Figs. 2** and **4**). For patients with a positive diagnosis of cancer or high grade dysplasia, esophagectomy [32–34] or endoscopic ablation or resection are inevitable. In such cases, PAE could assess the regional vasculature with high resolution, thereby assisting treatment planning or guiding the treatment procedure. Additionally, PAE’s capability of high-contrast, label-free transesophagel imaging of periesophageal blood vessels could enhance current pioneering EUS-based applications regarding the diagnosis, assessment, and treatment of many vascular diseases near the distal esophagus, such as gastroesophageal varices [35].

To realize the benefits of PAE in clinical settings, however, several technical issues should be addressed. Although we demonstrated PAE-based *in vivo* vascular imaging in the B-mode, seamless production of three-dimensional vasculature was limited by respiration motion artifacts. To produce continuous vascular maps *in vivo*, development and implementation of strategies to reduce respiratory motion artifacts is needed. Such strategies could include faster scanning speed [36], the addition of respiratory gating to the image acquisition, or the incorporation of an inflatable medical balloon [18,19] that limits tissue displacement. Another important technological development is to fully implement the functional imaging capability of PAE, which could enable early detection of disease symptoms. Although we utilized a single wavelength (584 nm) laser beam for this study, multi-wavelength PA imaging is also possible, as previously demonstrated [29]. Thus, transitioning to spectroscopic PA imaging would provide the clinician with more informative images and could increase the diagnostic accuracy of esophageal diseases. Additionally, PA-based quantitative metabolic rate measurements [37] could provide additional diagnostic information for disease screening [38,39].

## Conclusions

In this study, we acquired the first three-dimensional PA images of two rabbit esophagi, along with coregistered US images, using a dual-mode PA and US endoscopic probe. PAE provided high-resolution, three-dimensional images of microvasculature distributed in the walls of the esophagi and in the neighboring mediastinal regions. If motion artifacts can be resolved, we expect that this technique could be a valuable tool for assessing esophageal structure and function; diagnosing esophageal and periesophageal diseases, such as Barrett’s esophagus and gastroesohageal varices; and guiding various surgical procedures, such as esophagectomy.

## Supporting Information

S1 Fig Coregistered PAE-EUS pseudocolor images acquired from the esophagi *in vivo*. All RMAP images represent views from the inside of the esophagus. To artificially remove motion artifacts, we applied the spatial filtering to the mediastinal images only. In each image, the left- and right-hand sides correspond to the lower and upper esophagus, respectively, and the imaged area covers a 270° angular FOV (vertical) and a ∼12 cm long pullback distance (horizontal). In each axis, marks indicate the approximate mid-ventral (MV) and mid-dorsal (MD) positions, and the longitudinal location L0, where the carina is located. Scale bars, 10 mm (horizontal only).
(TIF)

S2 Fig Coregistered PAE-EUS pseudocolor *in vivo* and *ex vivo* RMAP images of the two rabbit esophagi. All the esophageal PAE and EUS images represent the raw Hilbert-transformed data without additional filtering (views from the inside of the esophagus). In each image, the left- and right-hand sides correspond to the lower and upper esophagus, respectively, and the imaged area covers a 270° angular FOV (vertical) and a ∼12 cm long pullback distance (horizontal). In each axis, marks indicate the approximate mid-ventral (MV) and mid-dorsal (MD) positions, and the longitudinal location L0, where the carina is located. Scale bars, 10 mm (horizontal only).
(TIF)

S3 Fig *In vivo* and *ex vivo* PA-RMAP images of the esophagi and mediastina of the two rabbits. All RMAP images represent views from the inside of the esophagus. To artificially remove motion artifacts, we applied the spatial filtering only to the mediastinal images. In each image, the left- and right-hand sides correspond to the lower and upper esophagus, respectively, and the imaged area covers a 270° angular FOV (vertical) and a ∼12 cm long pullback distance (horizontal). In each axis, marks indicate the approximate mid-ventral (MV) and mid-dorsal (MD) positions, and the longitudinal location L0, where the carina is located. Scale bars, 10 mm (horizontal only).
(TIF)

S4 Fig PAE-EUS three-dimensional image showing the mother blood vessel network. Presented are data from the longitudinal section, approximately from—L3.5 to L0 of **Fig. 4B**. In the right-hand side image, we artificially marked the mother blood vessel network. In each image, the *ρ*-axis corresponds to the radial depth, the *φ*-axis corresponds to the scanning mirror’s rotational direction, and the *z*-axis corresponds to the pullback direction.
(TIF)

S1 Video Three-dimensionally rendered PAE *ex vivo* image showing the vasculature of the esophageal wall of Rabbit 1 (corresponding to the data shown in Fig. 3G). The presented volumetric image was acquired over a ∼12 cm range with a ∼18 mm diameter.
(MOV)

S2 Video Serial PA and US *ex vivo* RMAP images of Rabbit 2 produced by changing the depth of removed surface signals. The presented images correspond to the data shown in **Fig. 4B and 4D**.
(MOV)

S3 Video Three-dimensionally rendered PAE-EUS pseudocolor *ex vivo* images showing vasculature and tissue density distribution in the mediastinal region of Rabbit 2. The presented volumetric images correspond to the data shown in **Fig. 4B and 4D** and cover a ∼18 mm diameter and ∼12 cm long image volume. The red and green colors correspond to PA and US signals, respectively, and the left-hand side of the image corresponds to the lower esophagus.
(MOV)
