# Supplementary figures and images for: Three-Dimensional Photoacoustic Endoscopic Imaging of the Rabbit Esophagus
Source: PLoS One. 2015 Apr 15;10(4):e0120269. doi: 10.1371/journal.pone.0120269 (PMC4398324; doi:10.1371/journal.pone.0120269)

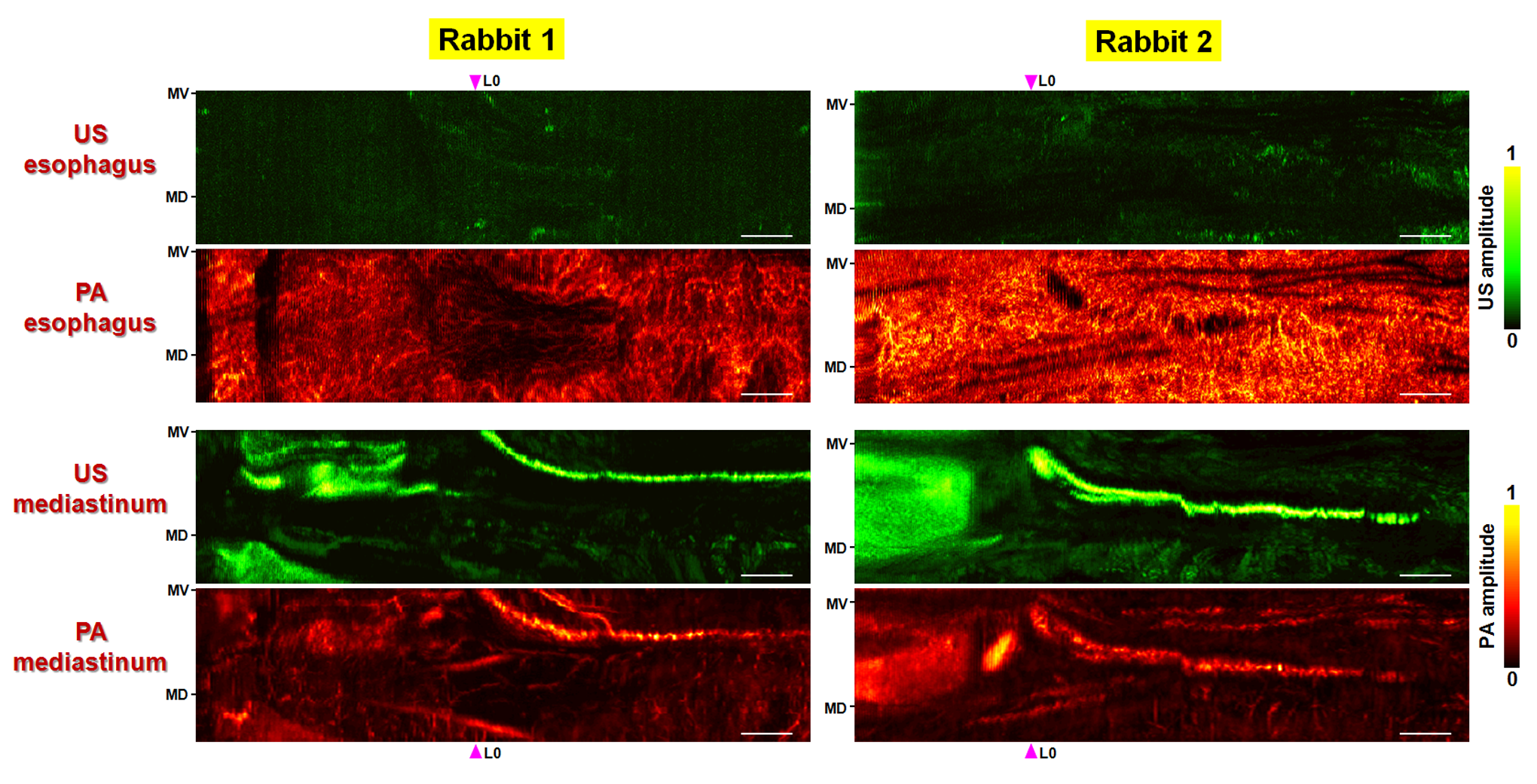

Supplement: S1 Fig — All RMAP images represent views from the inside of the esophagus. To artificially remove motion artifacts, we applied the spatial filtering to the mediastinal images only. In each image, the left- and right-hand sides correspond to the lower and upper esophagus, respectively, and the imaged area covers a 270° angular FOV (vertical) and a ∼12 cm long pullback distance (horizontal). In each axis, marks indicate the approximate mid-ventral (MV) and mid-dorsal (MD) positions, and the longitudinal location L0, where the carina is located. Scale bars, 10 mm (horizontal only). (TIF) [file pone.0120269.s001.tif]

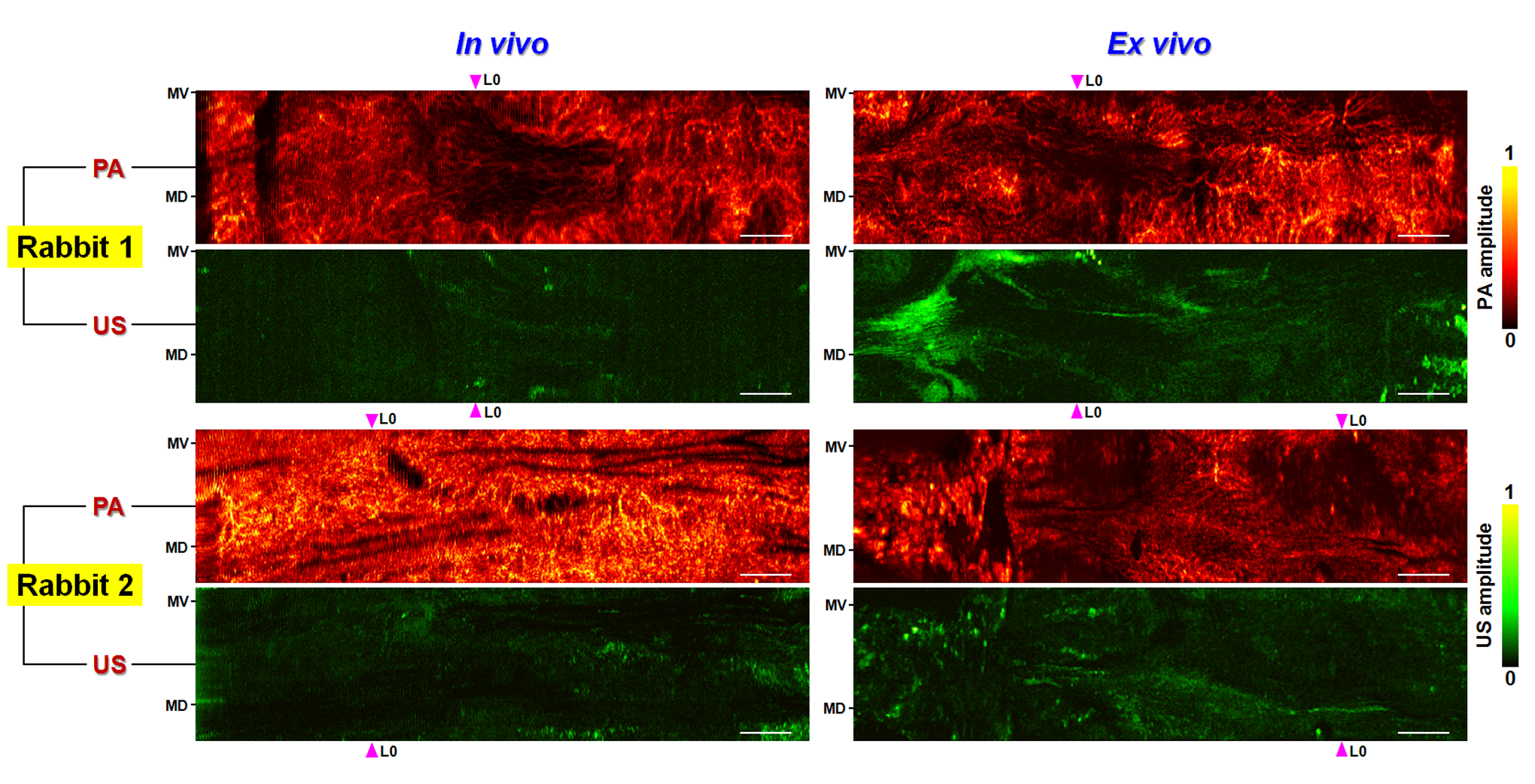

Supplement: S2 Fig — All the esophageal PAE and EUS images represent the raw Hilbert-transformed data without additional filtering (views from the inside of the esophagus). In each image, the left- and right-hand sides correspond to the lower and upper esophagus, respectively, and the imaged area covers a 270° angular FOV (vertical) and a ∼12 cm long pullback distance (horizontal). In each axis, marks indicate the approximate mid-ventral (MV) and mid-dorsal (MD) positions, and the longitudinal location L0, where the carina is located. Scale bars, 10 mm (horizontal only). (TIF) [file pone.0120269.s002.tif]

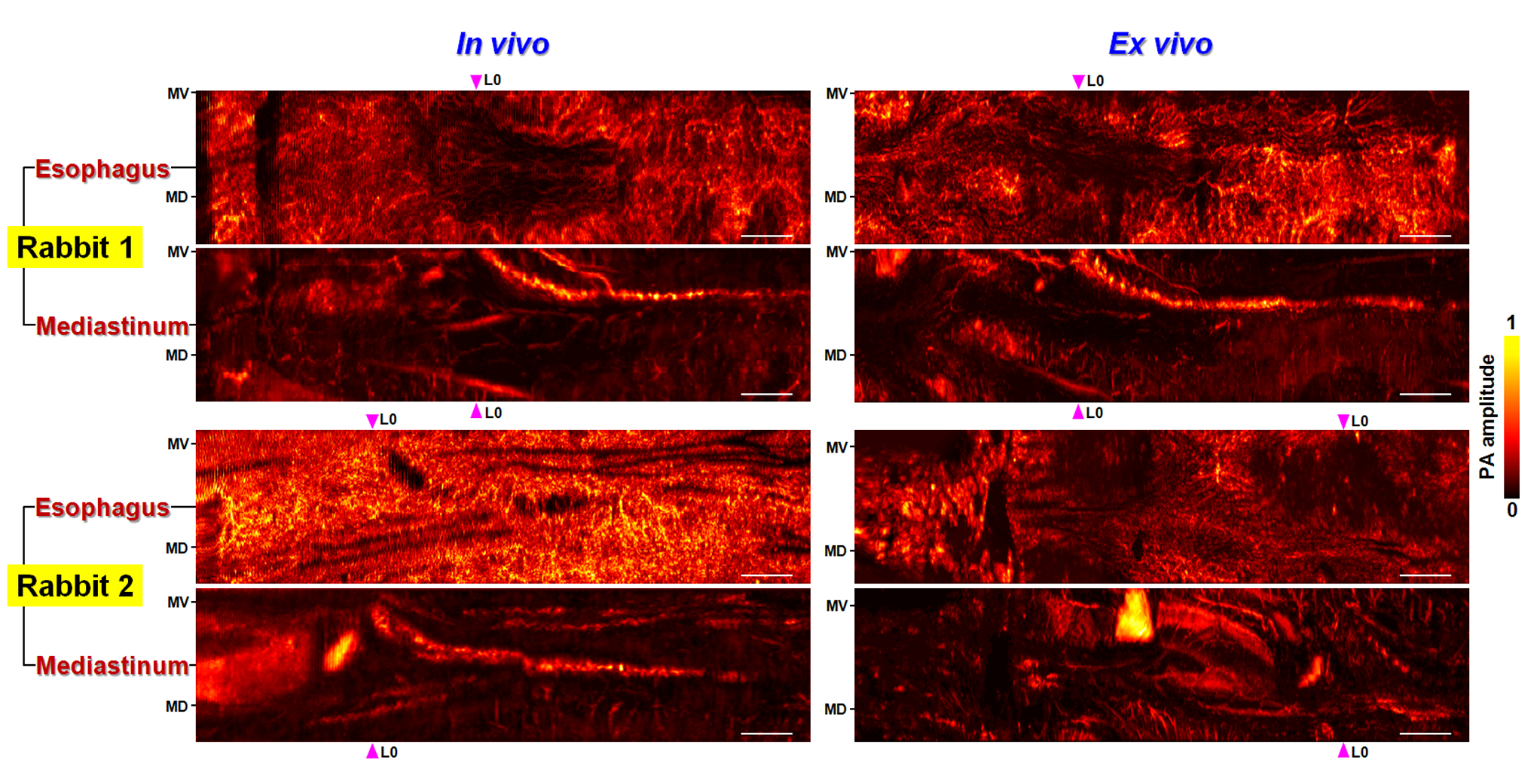

Supplement: S3 Fig — All RMAP images represent views from the inside of the esophagus. To artificially remove motion artifacts, we applied the spatial filtering only to the mediastinal images. In each image, the left- and right-hand sides correspond to the lower and upper esophagus, respectively, and the imaged area covers a 270° angular FOV (vertical) and a ∼12 cm long pullback distance (horizontal). In each axis, marks indicate the approximate mid-ventral (MV) and mid-dorsal (MD) positions, and the longitudinal location L0, where the carina is located. Scale bars, 10 mm (horizontal only). (TIF) [file pone.0120269.s003.tif]

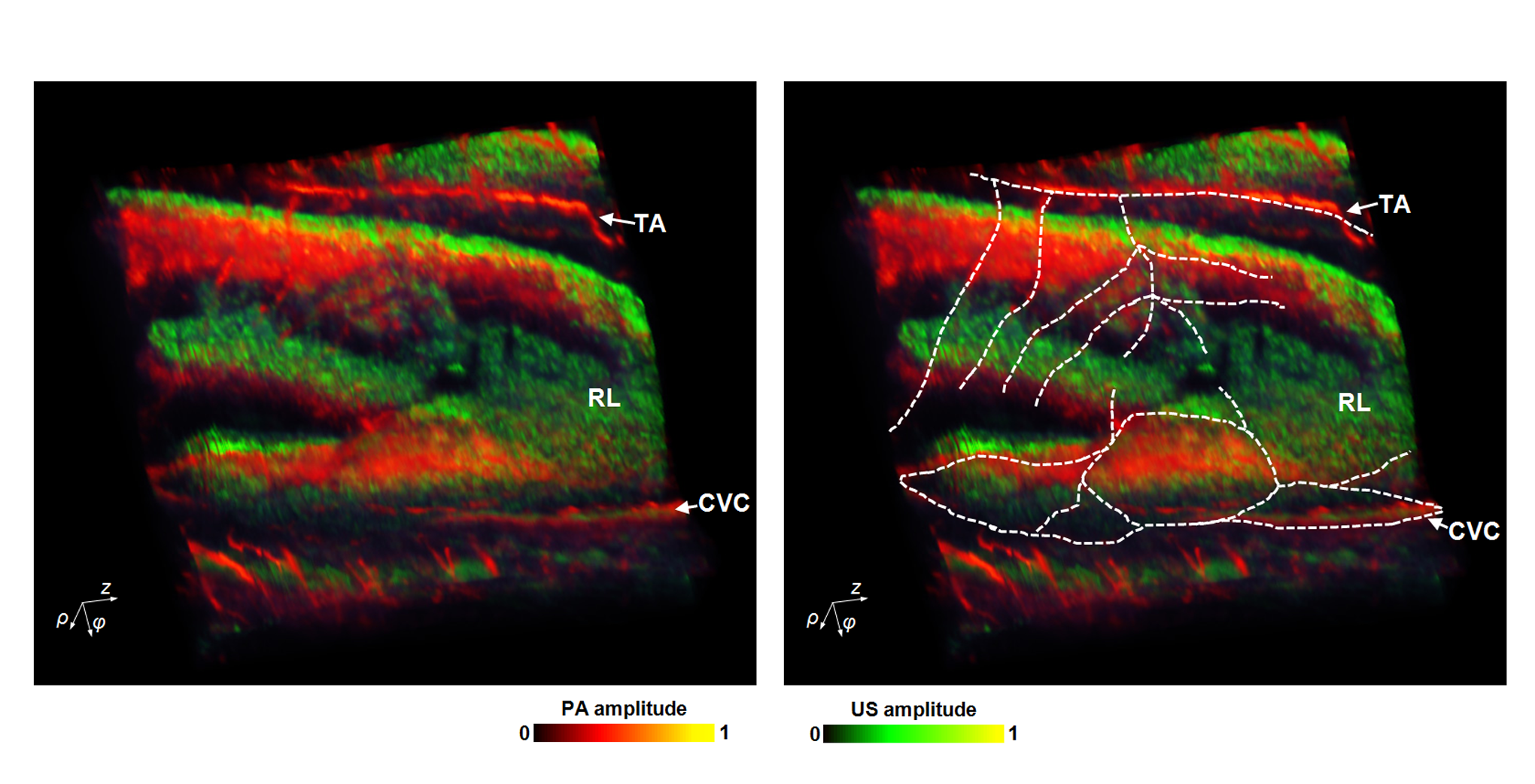

Supplement: S4 Fig — Presented are data from the longitudinal section, approximately from—L3.5 to L0 of Fig. 4B. In the right-hand side image, we artificially marked the mother blood vessel network. In each image, the ρ-axis corresponds to the radial depth, the φ-axis corresponds to the scanning mirror’s rotational direction, and the z-axis corresponds to the pullback direction. (TIF) [file pone.0120269.s004.tif]
